# Endogenously Generated Plasmin at the Vascular Wall Injury Site Amplifies Lysine Binding Site-Dependent Plasminogen Accumulation in Microthrombi

**DOI:** 10.1371/journal.pone.0122196

**Published:** 2015-03-25

**Authors:** Tomasz Brzoska, Aki Tanaka-Murakami, Yuko Suzuki, Hideto Sano, Naohiro Kanayama, Tetsumei Urano

**Affiliations:** 1 Department of Medical Physiology, Hamamatsu University School of Medicine, Hamamatsu, Japan; 2 Department of Gynecology, Hamamatsu University School of Medicine, Hamamatsu, Japan; Baker IDI Heart and Diabetes Institute, AUSTRALIA

## Abstract

The fibrinolytic system plays a pivotal role in the regulation of hemostasis; however, it remains unclear how and when the system is triggered to induce thrombolysis. Using intra-vital confocal fluorescence microscopy, we investigated the process of plasminogen binding to laser-induced platelet-rich microthrombi generated in the mesenteric vein of transgenic mice expressing green fluorescent protein (GFP). The accumulation of GFP-expressing platelets as well as exogenously infused Alexa Fluor 568-labeled Glu-plasminogen (Glu-plg) on the injured vessel wall was assessed by measuring the increase in the corresponding fluorescence intensities. Glu-plg accumulated in a time-dependent manner in the center of the microthrombus, where phosphatidylserine is exposed on platelet surfaces and fibrin formation takes place. The rates of binding of Glu-plg in the presence of ε-aminocaproic acid and carboxypeptidase B, as well as the rates of binding of mini-plasminogen lacking kringle domains 1-4 and lysine binding sites, were significantly lower than that of Glu-plg alone, suggesting that the binding was dependent on lysine binding sites. Furthermore, aprotinin significantly suppressed the accumulation of Glu-plg, suggesting that endogenously generated plasmin activity is a prerequisite for the accumulation. In spite of the endogenous generation of plasmin and accumulation of Glu-plg in the center of microthrombi, the microthrombi did not change in size during the 2-hour observation period. When human tissue plasminogen activator was administered intravenously, Glu-plg further accumulated and the microthrombi were lysed. Glu-plg appeared to accumulate in the center of microthrombi in the early phase of microthrombus formation, and plasmin activity and lysine binding sites were required for this accumulation.

## Introduction

The fibrinolytic system plays a central role in thrombolysis, and it is basically composed of two steps. First, plasmin is generated by proteolytic cleavage of its zymogen form (plasminogen) by plasminogen activators (PAs). Second, the insoluble fibrin is digested by the generated plasmin [1]. The system is finely regulated at both steps by many components, including specific inhibitors of the corresponding enzymes, in order to achieve a balance between avoiding premature dissolution of hemostatic thrombi and facilitating lysis of excessive thrombi. Each step of the underlying mechanisms of these reactions has been well characterized. However, it is not fully clear how and when the system is triggered to start dissolving the thrombus. In the present study, we employed an *in vivo* imaging system to analyze how the components involved in fibrinolysis interact within the thrombus.

Glu1-plasminogen (Glu-plg) consists of an N-terminal Pan-apple domain, 5 repeats of highly homologous kringle domains having triple disulfide-linked peptide regions composed of 80 to 90 amino acid residues each, and a serine protease domain [1]. The kringle domains contain lysine-binding sites (LBSs) that play essential roles in binding to ligands with lysine residues at their C-termini, such as partially degraded fibrin or plasminogen-binding cell surface proteins [1]. LBSs also play a role in maintaining the proper conformation of Glu-plg through intra-molecular interactions. Glu-plg adopts a tightly closed conformation in plasma through intra-molecular binding of lysine 50 in the Pan-apple domain to the LBS in kringle 5 [2]. The LBS in kringle 1 is accessible to the C-terminal lysine even in its closed form, and thus it is considered to be the initial binding site. As a consequence of further binding to additional C-terminal lysines and release of the Pan-apple domain from the LBS in kringle 5, Glu-plg adopts an open conformation that can be more readily activated by PAs [2]. Tissue plasminogen activator (tPA), which is the main PA in the circulation and which has a strong affinity to fibrin, effectively activates plasminogen bound on the fibrin surface by forming a tri-molecular complex following its exocytosis from vascular endothelial cells (VECs) [3]. Recently we have shown that Glu-plg accumulates on VECs and the surface of fibrin formed on VECs in an LBS-dependent and plasmin activity-dependent manner, and these events precede effective fibrinolysis on VECs [4]. Thus, the binding of Glu-plg on the surface of fibrin appears to be important, as it initiates fibrinolysis.

We recently reported that the extent of platelet activation is uneven within microthrombi generated in a murine model, and that platelets expressing phosphatidylserine (PS) on their surface exist only in the center of the thrombus [5]. As a consequence of the activation of vitamin-K dependent coagulation factors on the exposed PS [6], fibrin formation was also clearly demonstrated to take place only in the center of the thrombus. Such uneven distribution of both PS-bearing platelets and fibrin in the thrombus seems to be important not only in stabilizing the thrombus but also in limiting its size. A similarly layered thrombus structure was also recently demonstrated by others [7]. In the present study, we targeted Glu-plg to be visualized in microthrombi formed in an *in vivo* system where both injured and intact VECs exist in close proximity. Both time- and space-dependent accumulation of Alexa Fluor 568-labeled Glu-plg (Glu-plg-568) were visualized in green fluorescent protein (GFP)-expressing transgenic mice (GFP-mice). This accumulation was LBS-dependent and required endogenously generated plasmin activity.

## Materials and Methods

### Animals

Transgenic C57BL/6J mice expressing green fluorescence protein (GFP mice) and wild-type (WT) C57BL/6J mice were obtained from SLC (Shizuoka, Japan). This study was carried out in strict accordance with the recommendations in the Japanese guidelines and regulations for scientific and ethical animal experimentation. The experimental protocol for the study was reviewed and approved by the Animal Experiments Committee of Hamamatsu University School of Medicine (permit number: 2009030). All surgery was performed under sodium pentobarbital anesthesia, and all efforts were made to minimize suffering.

### Proteins and chemicals

Glu-plg was purified from freshly frozen human plasma by affinity chromatography on Sepharose-lysine, and mini-plasminogen (mini-plg) was prepared by elastase digestion of Glu-plg followed by chromatography on Sepharose-lysine and elution with a gradient of ε-aminocaproic acid (EACA) [8]. Proteins were labeled by Alexa Fluor 568 and 488, purchased from Molecular Probes (Eugene, OR, USA), and used according to the manufacturer’s instructions. Alexa Fluor 568-labeled Glu-plg (Glu-plg-568; dye/protein molar ratio = 4.0) was similarly activated by recombinant tissue plasminogen activator (tPA) to non-labeled Glu-plg in the presence of poly-D-Lys [9], and bound to Sepharose-lysine in a similar manner as non-labeled Glu-Plg (data not shown). EACA, carboxypeptidase B (CPB), aprotinin, and ionomycin (IMC) were purchased from Sigma (St. Louis, MO, USA) and human thrombin was purchased from Mitsubishi Tanabe Pharma Co. (Osaka, Japan). Annexin A5 (ANX) and recombinant tPA (Alteplase) were provided by KOWA Pharmaceuticals (Tokyo, Japan) and Genentech Inc. (South San Francisco, CA, USA), respectively.

### *In vivo* imaging experiments

For intra-vital confocal fluorescence microscopy to observe the microcirculation in a living mouse, we used a Yokogawa Real-Time 3D Workstation composed of a Nikon TE 600 microscope (×20, NA 0.5 or ×40, an NA 0.8 water immersion objective; Nikon Corporation, Tokyo, Japan), a Yokogawa CSU-21 confocal scanner unit (Yokogawa Electric Corporation, Tokyo, Japan), an electron-bombarded (EB)–CCD (Hamamatsu Photonics, Hamamatsu, Japan), and a piezoelectric driver (P721.17; Physil Instrumente GmbH and Co. KG, Karlsruhe, Germany), with which a focal plane image (one optical section) can be taken at 33 ms along the z axis. GFP or Alexa Fluor 488 and Alexa Fluor 568 were simultaneously excited by 488 and 568 nm lasers (Krypton Argon ion lasers; 643-YB-A01; Melles Griot Laser Group, Carlsbad, CA, USA). For the simultaneous monitoring of both fluorescent wavelengths, an emission beam splitter, a W-view Optics unit (A 4313–11; Hamamatsu Photonics, Hamamatsu, Japan), was installed between the scanner unit and the CCD. The W-view consisted of a dichroic mirror of 550 nm and two emission filters, a 510/23 nm band pass filter for GFP and Alexa Fluor 488 and a 590 nm long pass filter for Alexa Fluor 568, so that two separate images of GFP or Alexa Fluor 488 and Alexa Fluor 568 fluorescence could be produced simultaneously.

### Laser-induced vessel wall injury in GFP or WT mice

After being lightly pre-anesthetized with diethyl ether, mice (approximately 20 g body weight each) were anesthetized by intraperitoneal injection of Nembutal (50 mg/kg; Dainippon Pharma, Osaka, Japan). A midline laparotomy incision was made, and then the mesentery of the ileum was pulled out of the abdomen and draped over a plastic mound. The mesentery was continuously perfused with saline at 37°C to prevent the blood vessels from drying out. A 100 μl aliquot of 1.5 μM Glu-plg-568 was injected through the tail vein of the GFP mice 10 minutes before the laser injury. When needed, EACA (200 mM, 100 μl), CPB (100 U/ml, 100 μl), aprotinin (10.000 U/ml, 50 μl) or the corresponding volumes of 0.9% NaCl as a control were administered 5 minutes before the injection of Glu-plg-568. In another set of experiments, Alexa Fluor 568-labeled mini-plg (mini-plg-568; dye/protein molar ratio = 2.3) was injected instead of Glu-plg-568. The spatial distribution of Glu-plg-568 within the microthrombus was examined by means of Alexa Fluor 488-labeled Annexin A5 (ANX-488; 2 μg/g mouse body weight) administration 5 minutes before Glu-plg-568 into the tail vein of the WT mice.

Mesenteric venules were identified and endothelial injury was induced by irradiation with a 514-nm argon-ion laser (543-GS-A03; Melles Griot Laser Group, Carlsbad, CA, USA). The laser beam was aimed at the endothelium through the microscope objective lens. The area of laser-induced injury on the endothelium, the diameter of which was approximately 10 μm, was kept constant by changing the intensity and duration of laser irradiation [5]. To reduce the numbers of mice to be sacrificed, at most two thrombi were formed in some experiments at an approximately 5 minute interval, and images of the corresponding thrombus were taken at the indicated time points.

After each experiment, the anesthetized animals were euthanized by cervical dislocation.

### Image analysis

A z-stack of 40 optical sections at up to 30 frames per second from the vessel wall to the luminal surface of a thrombus was captured, starting from the fifth second, for up to 2 hours after laser injury (1 μm optical slice thickness, 40 z-sections collected at 1 μm intervals) and analyzed using a Yokogawa Real-Time 3D Workstation and IPLab software (BD Biosciences Bioimaging, MD). A freehand-defined region of interest was traced along the outline of fluorescent areas and the integrated fluorescence was calculated. Areas of the corresponding thrombi optical sections were calculated by using Adobe Photoshop CS5. When needed, the fluorescence intensity of GFP was normalized to the initial value of the experiment, whereas that of Glu-plg-568 was normalized to the maximal value from the experiment with Glu-plg-568 alone. To obtain the perpendicular plane of merged images of the thrombus, sequential focal plane images (optical sections) of GFP and Glu-plg-568 fluorescence were merged. These were then reconstructed into 3D images of a thrombus using VoxBlast 3.1 (VayTek Inc. Fairfield, IA, USA).

### Thrombolysis by exogenously infused tPA

Forty minutes after the injury, tPA (3 mg/kg) or an equivalent volume of 0.9% NaCl was administered through the femoral vein of GFP mice. EACA (4.25 mmol/kg) or an equivalent volume of 0.9% NaCl was administered through the other femoral vein 10 minutes before each injury when necessary. Images were collected 2 minutes before and every 5 minutes after tPA administration for 1 hour. VoxBlast 3.1 was used to obtain 3D images of the thrombi. Areas of 1 μm thick thrombi optical sections were determined by employing Adobe Photoshop CS5. Further, volumes of thrombi were obtained by summing up the calculated volumes of thrombi optical sections.

In another set of experiments, Glu-plg-568, at the same dose as described earlier, was administered 10 minutes before each laser injury. Forty minutes after microthrombus formation, either tPA or an equivalent volume of 0.9% NaCl was infused. Images were collected as described above, and additional images were also captured every 5 minutes after laser injury. A freehand-defined region of interest was traced along the outline of the fluorescent areas, and integrated fluorescence intensities associated with the Glu-plg-568 were calculated. In addition, each fluorescence intensity was normalized to the value obtained 2 minutes before administration of either tPA or saline.

### *In vitro* imaging experiments

#### Platelet preparation

Blood samples were collected through the inferior vena cava of anesthetized GFP mice with a syringe containing 1/10 of their total blood volume of 3.8% trisodium citrate. Platelet rich plasma (PRP) was prepared by centrifugation at 100×g for 10 min at 22°C. When needed, PRP was further centrifuged at 700×g for 10 min at 22°C, and the precipitated platelets were washed twice and resuspended in 10 mM HEPES buffer, pH 7.4, containing 140 mM NaCl, 5 mM KCl, 1 mM MgCl_2_, 10 mM glucose, and 1 mM pyruvate (washed platelets) at the required concentration. Anesthetized mice were euthanized by cervical dislocation.

### Fluorescence confocal laser scanning microscopy study

A confocal laser scanning microscope (CLSM) (FV1000; Olympus) equipped with a 100× (NA 1.40) oil immersion objective lens and a temperature control system was used. All experiments were conducted in 35 mm glass bottom dishes at 37°C [10]. Suspensions of washed platelets (100 μl, 2×10^4^ platelets/μl) were supplemented with non-labeled Glu-plg (0.45 μM), Glu-plg-568 (50 nM), and EACA (100 mM) or CPB (15 U/ml) as needed, and were transferred onto glass bottomed dishes to allow platelets to adhere for 20 minutes at room temperature. Each experiment commenced when CaCl_2_ (0.5 mM) and IMC (10 μM) were added to activate the platelets in a total volume of 200 μl. In another set of experiments, platelet rich plasma (2×10^4^ platelets/μl) that had been diluted twice with 0.9% NaCl was employed, and fibrin network formation was initiated by addition of CaCl_2_ (10 mM) and thrombin (1 U/ml) in a total volume of 200 μl [10]. Immediately after either IMC or thrombin addition, at a randomly chosen location with the focal plane being approximately either 1 μm (in the absence of a fibrin network) or 3 μm (in the presence of a fibrin network) above the bottom of the dish, images were taken every 30 s for 120 minutes. The calculated optical thickness of each slice was 0.93 μm. Collected images were analyzed using FV10-ASW (Olympus) and AquaCosmos 2.6 (Hamamatsu Photonics, Hamamatsu, Japan) software. The platelet areas were calculated by using Adobe Photoshop CS5.

### Statistical analysis

Repeated measures ANOVA models were used to analyze the modification of kinetics of Glu-plg-568 accumulation within microthrombi. The significance of differences between the two groups was evaluated by means of a *t-*test for independent samples.

## Results

### The accumulation of Glu-plg in microthrombi in an in vivo system

**Fig 1:**
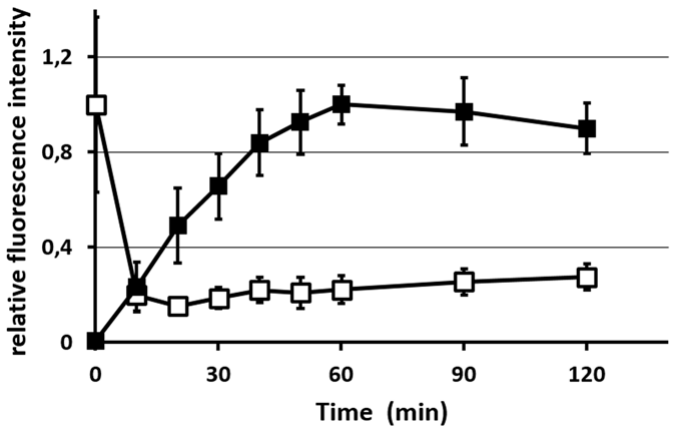
The kinetics of Glu-plg-568 accumulation at a site of laser injury. Relative changes in the fluorescence intensity of GFP (open squares) and Glu-plg-568 (closed squares) in the horizontal (X-Y) images after laser injury. Results are expressed as relative fluorescence intensity compared to the maximum value measured (relative value of 1) at the corresponding fluorescence (the initial value of the GFP signal and the maximal value of the Glu-plg-568 signal). Each point represents the mean value of 5 thrombi (from 5 mice) ± standard deviation (SD).

**Fig 2:**
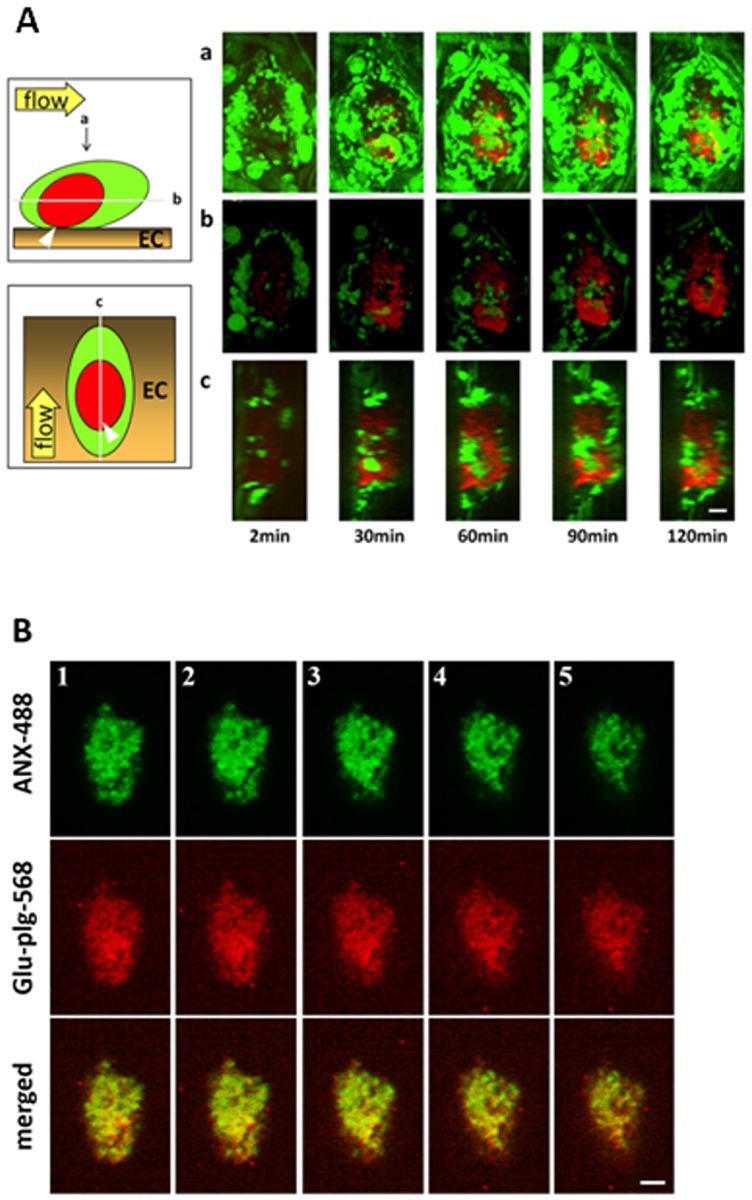
Spatiotemporal distribution of Glu-plg-568 within the microthrombus. (A) Localization of Glu-plg-568 in a microthrombus in a GFP mouse. a) images of the whole thrombus from the luminal side. b) horizontal plane (X-Y) images at the point where the area of Glu-plg-568 was the largest. c) perpendicular plane (Y-Z) images at the point of the longest Y- axis. Images were taken at 2, 30, 60, 90, and 120 min after laser injury. The schematic depiction shows the horizontal and vertical planes of a laser-induced thrombus containing Glu-plg-568 (red) and GFP platelet (green) on the injured endothelial cells (EC). Glu-plg-568 accumulated in the center of the microthrombus in a time-dependent manner. The arrow shows the direction of blood flow. Scale bar, 10 μm. (B) Co-localization of the PS (ANX-488) and Glu-plg-568 within the microthrombus. ANX-488 and Glu-plg-568 were injected into the tail vein of WT mice before laser injury. Images were collected 60 minutes after the mesentery injury. Five optical sections (1–5) were selected at 2-μm intervals from the vessel wall (1) to the luminal surface of a thrombus (5) to examine the spatial distribution of PS-exposing platelets and Glu-plg. Localization of Glu-plg-568 within the microthrombus paralleled the localization of ANX-488 bound to the surface of PS-expressing platelets (n = 3 thrombi from three mice). Scale bar, 10 μm.

Platelet-rich microthrombus formation was visualized by noting the accumulation of GFP-expressing platelets at the laser-induced injury site, as described previously [5] [11]. Glu-plg-568, here used only as a marker in the physiological process of fibrinolysis, accumulated in microthrombi in a time-dependent manner within 1 hour after laser injury (Fig. 1). The accumulation occurred mainly in the center of the thrombi, where GFP fluorescence intensity decreased after an initial increase. The decrease in fluorescence intensity of GFP from the platelets in the center of the thrombi was followed by exposure of PS on their surfaces. A similar decrease in GFP fluorescence intensity followed by PS exposure was shown in an *in-vitro* system for platelets that expressed GFP after ionomycin treatment [5]. Thus, GFP likely leaked from fully activated platelets due to alterations in membrane permeability [5]. The spatial distribution of Glu-plg-568 in the thrombi was probed using a piezoelectric driver that enabled collection of focal plane images along the z axis [5]. The areas of Glu-plg-568 accumulation in the center of the thrombi extended both to the luminal side and downstream of the blood flow, as shown in the sagittal and horizontal reconstructed figures, respectively (Fig. 2A). ANX-488 and Glu-plg-568 administered to the WT mice before each laser injury clearly co-localized within the microthrombus, indicating that Glu-plg-568 accumulated at a site where platelets expose PS on their surfaces (Fig. 2B).

### Glu-plg bound to microthrombi via an LBS-dependent mechanism

**Fig 3:**
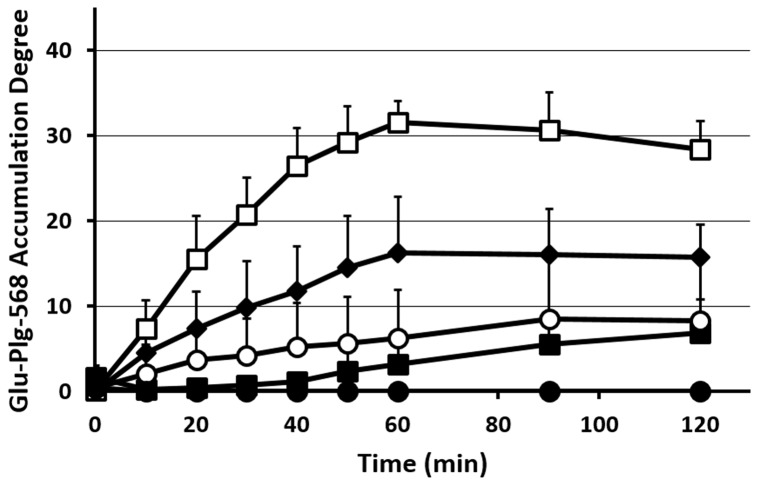
Changes in Glu-plg-568 accumulation in microthrombi after laser injury. The kinetics of Glu-plg-568 accumulation are shown as an increase in the integrated fluorescence intensity of Glu-plg-568 per corresponding thrombus area in the same optical section 60 minutes after laser-induced thrombus formation. Reagents were EACA (closed squares, N = 5, 5 thrombi from 3 mice, p < 0.001), CPB (closed diamond, N = 5, 5 thrombi from 5 mice, p < 0.005) and aprotinin (open circles, N = 4, 4 thrombi from 2 mice, p < 0.001). Control experiments are shown as open squares (N = 5, 5 thrombi from 5 mice) and mini-plg-568 as closed circles (N = 4, 4 thrombi from 4 mice, p < 0.001). This assay was analyzed with repeated measures ANOVA. Each point represents the mean ±SD; N = number of thrombi.

To evaluate the involvement of LBS in the accumulation of Glu-plg in the microthrombi, we treated GFP mice before laser injury with EACA and Glu-plg-568 and monitored the accumulation of Glu-plg-568. The amount of Glu-plg-568 that accumulated in the thrombi of mice treated with EACA was significantly less than that measured in control mice (Fig. 3). Pretreatment with carboxypeptitase B (CPB), which eliminates Lys- and Arg-residues at the C-termini of proteins, an action similar to that of thrombin-activatable fibrinolysis inhibitor (TAFI) [12], also suppressed the accumulation of Glu-plg-568 in the thrombi (Fig. 3). These results suggest that the accumulation of Glu-plg is LBS-dependent. This was further confirmed by the fact that mini-plg-568 (Val443-Asn791), which is composed of kringle 5 and the protease domain and does not possess LBS, accumulated at only negligible levels at the center of the microthrombus (Fig. 3).

Plasmin is a chymotrypsin-type serine protease and cleaves peptide bonds with either a Lys- or Arg- residue at its P1 position, thereby exposing lysine at the C-terminus of the substrate molecule following limited proteolysis [1]. In order to determine the role of the C-terminal lysine, which is newly exposed via the action of endogenously generated plasmin, we employed aprotinin, a serine protease inhibitor that strongly inhibits the enzymatic activity of plasmin. Injection of aprotinin prior to vascular wall injury significantly diminished the accumulation of Glu-plg-568 in microthrombi (Fig. 3). This suggests that the C-terminal lysine to which Glu-plg bound was produced by proteolytic cleavage of proteins by plasmin in the thrombi. It also suggests that plasmin is generated within the microthrombi at an early phase after vascular injury.

### Glu-plg binds to the activated platelet surface *in vitro*

**Fig 4:**
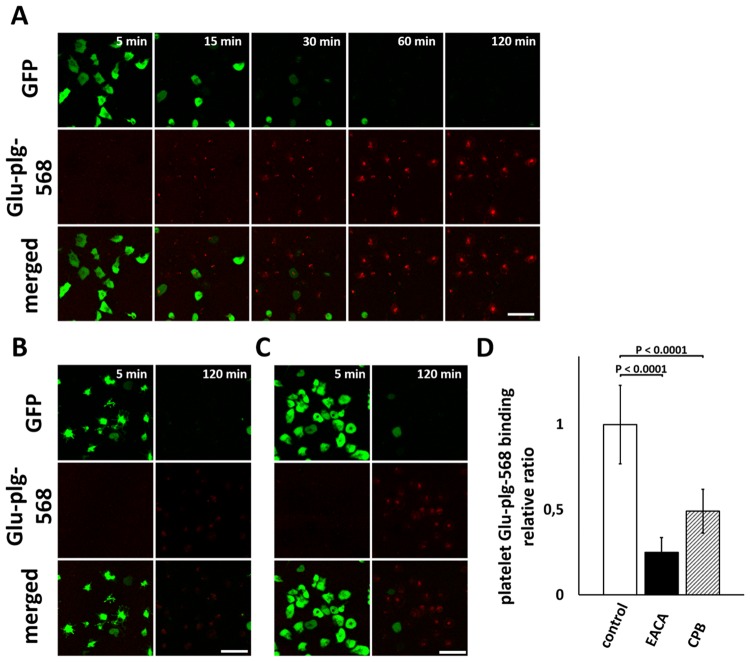
Time-dependent accumulation of Glu-plg-568 on the surface of IMC-treated washed platelets from GFP-mice. Washed platelets from GFP-mice were treated with IMC (10 μM) in the presence of Glu-plg-568, and accumulation of Glu-plg-568 on the surface of platelets was monitored over time by CLSM either in the absence (A) or in the presence of 100 mM EACA (B) or 15 U/ml CPB (C). Representative CLSM images are shown at the indicated time points after addition of IMC. A decrease in GFP fluorescence intensity in individual platelets indicates the full activation of platelets with PS exposure on the surface, as a result of a sustained elevation of intracellular calcium ion concentration caused by IMC. The decrease in GFP fluorescence intensity was followed by gradual binding of Glu-plg-568 to the platelet surfaces, which was strongly limited by EACA and CPB. The scale bars represent 10 μm. (D) The platelet Glu-plg-568 binding relative ratio was expressed as the integrated fluorescence intensity of Glu-plg-568 bound to the surface of activated platelet 120 minutes after IMC supplementation and divided by the area of the platelet (n = 30 activated platelets from three independent experiments in each column). This assay was analyzed with a *t*-test for independent samples. Results are normalized to the control sample mean value (mean ± SD).

Glu-plg-568 accumulated in the center of the thrombi, where platelets are fully activated, where they expose PS and where fibrin is formed [5]. We thus investigated whether Glu-plg binds to the surface of platelets activated *in vitro*. When the washed platelets obtained from GFP-mice were treated with 10 μM IMC in the presence of CaCl_2_ and Glu-plg-568, progressive activation of individual platelets was observed, as evidenced by a sudden decrease in GFP fluorescence intensity measured by CLSM, followed by a gradual increase in Glu-plg-568 binding to the platelet surfaces (Fig. 4A).

**Fig 5:**
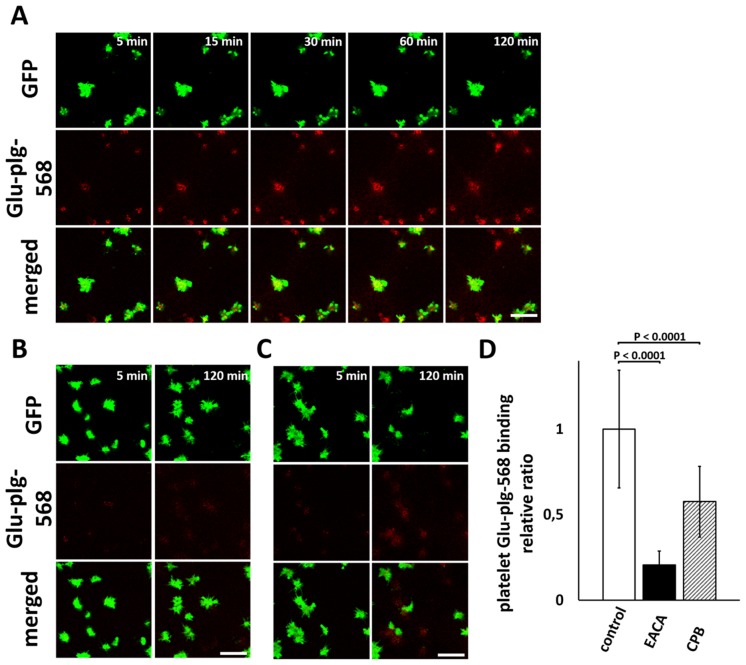
Time-dependent accumulation of Glu-plg-568 on the platelet surfaces from GFP-mice incorporated into the fibrin network. Diluted PRP from GFP-mice was treated with thrombin (1 U/ml) in the presence of Glu-plg-568, and accumulation of Glu-plg-568 on the surface of platelets incorporated into the fibrin network was monitored over time by CLSM, either in the absence (A) or in the presence (B) of 100 mM of EACA or 15 U/ml CPB (C). Representative CLSM images were obtained 3 μm from the bottom of the dish at the indicated time points after addition of thrombin (1 U/ml). Fibrin network formation and the incorporation of platelets in the network were clearly demonstrated after addition of thrombin, which was followed by accumulation of Glu-plg-568 on the surface of platelets even prior to the decrease in GFP fluorescence intensity. Binding of Glu-plg-568 was significantly inhibited in the presence of EACA and CPB. The scale bars represent 10 μm. (D) The platelet Glu-plg-568 binding relative ratio was expressed as the integrated fluorescence intensity of Glu-plg-568 bound to the surface of platelet 120 minutes after thrombin supplementation and divided by the area of the platelet (n = 30 activated platelets from three independent experiments in each column). This assay was analyzed with a *t*-test for independent samples. Results are normalized to the control sample mean value (mean ± SD).

Both EACA (100 mM) and CPB (15 U/ml) showed large decreases in platelet binding of Glu-plg-568 (Fig. 4B, C, D). Thus, the surface of platelets appears to be a possible source of C-terminal lysines to which Glu-plg binds when platelets are extensively activated. In another set of experiments, we investigated how Glu-plg binds to the platelet surface in the presence of fibrin(ogen). Consistent with earlier results, treatment of diluted PRP with thrombin and CaCl_2_ irreversibly evoked fibrin network formation and the binding of platelets to fibrin(ogen) [10]. Under these conditions, Glu-plg-568 accumulated in a time-dependent manner to the surface of platelets that were not fully activated and that still contained GFP. This accumulation was also limited by EACA (100 mM) and CPB (15 U/ml) (Fig. 5). Together, these results suggested that Glu-plg-568 bound to the surface of platelets that were either fully activated to express PS or that were not fully activated but that still bound to fibrin(ogen).

### Dissolution of microthrombi after tPA infusion

**Fig 6:**
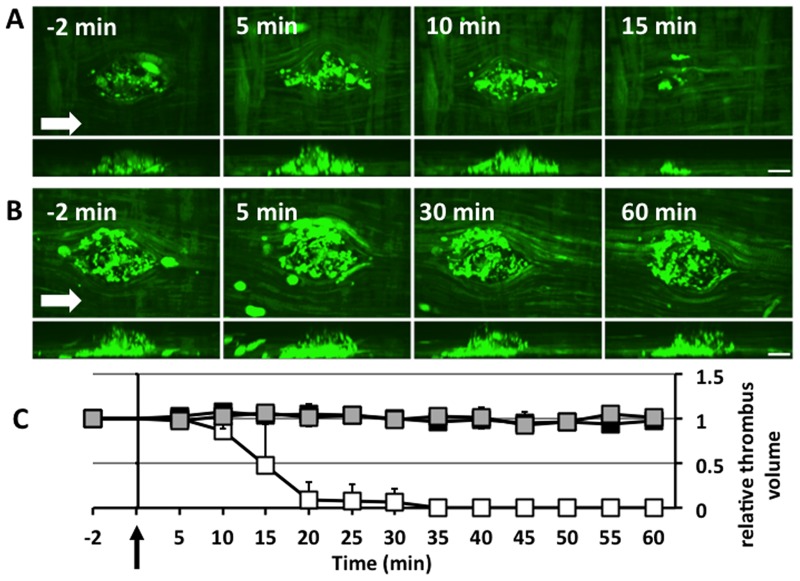
tPA-induced lysis of microthrombi. (A) tPA was injected at a dose of 3 mg/kg 40 minutes after the laser-induced injury. Images were captured 2 minutes before and every 5 minutes up to one hour after tPA administration. (B) In another set of experiments, EACA (4.25 mmol/kg) was administered intravenously through the femoral vein of GFP-mice 10 minutes before the laser injury, and images were captured in a similar manner. In both Figures (A) and (B), the upper panel shows horizontal plane (X-Y) images and the lower panel shows perpendicular plane (Y-Z) images, which were reconstructed from sequential optical sections of microthrombi. Arrows show the direction of blood flow. The scale bars represent 10 μm. (C) Relative changes in the volumes of thrombi after tPA administration. tPA or saline was injected at time 0 (arrow). Volumes of thrombi were normalized to their values 2 minutes before saline or tPA injection. Thrombi in mice treated with saline did not change in size (gray squares), whereas all of the thrombi in mice treated with tPA 40 minutes after the laser injury dissolved completely within 35 minutes after tPA administration (white squares). When mice were pretreated with EACA 10 minutes before the laser injury, however, tPA-dependent thrombolysis was totally inhibited (black squares). Each point represents the mean value of 5 thrombi from 5 mice ± SD.

**Fig 7:**
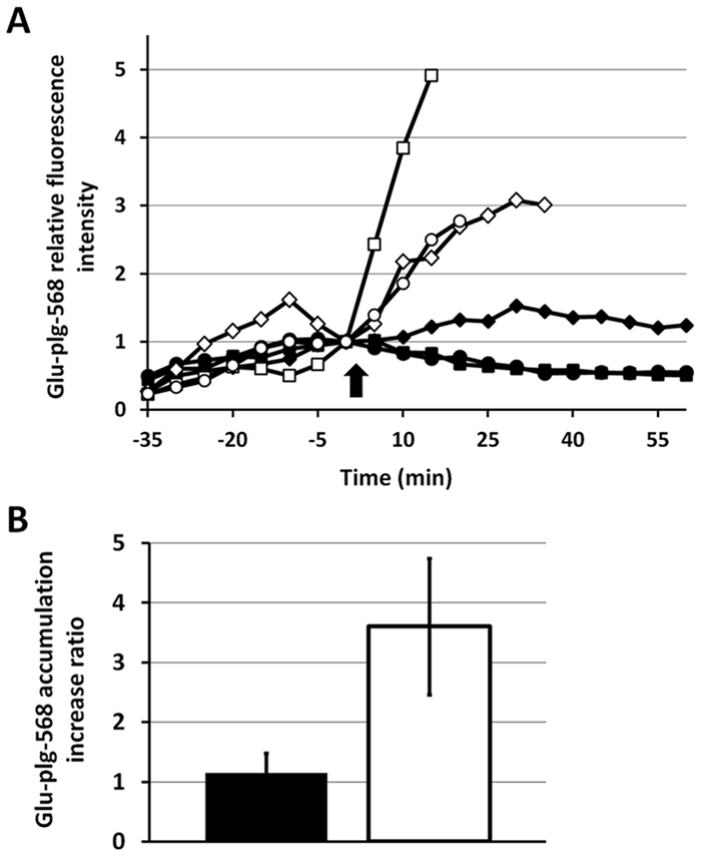
Changes in Glu-plg-568 relative fluorescence intensity induced by exogenous tPA administration. (**A**) Glu-plg-568 was administered to GFP-mice 10 minutes before each laser injury and 40 minutes after either human tPA (white: square, diamond, circle; three individual experiments) or an equivalent volume of 0.9% NaCl (black: square, diamond, circle; three individual experiments) was infused. Thrombi images were collected every 5 minutes after the laser-evoked endothelial injury, starting 2 minutes before and then every 5 minutes up to 60 minutes after either tPA or saline infusion. Each fluorescence intensity was normalized to the value obtained 2 minutes before either tPA or saline administration. tPA or saline was injected at time 0 (arrow). (B) Shown are the Glu-plg-568 accumulation increase ratios, which were expressed as the average values of the maximum increases in Glu-plg-568 relative fluorescence intensity after administration of either tPA (mean ± SD, n = 3, white column) or saline (mean ± SD, n = 3, black column). This assay was analyzed with a *t*-test for independent samples (*P* < 0.05).

Although Glu-plg accumulated in the thrombi, in which plasmin played an important role, the microthrombi did not dissolve spontaneously within 2 hours. To determine whether the microthrombus was sensitive to fibrinolysis, tPA (3 mg/kg) was given intravenously 40 minutes after thrombus formation. tPA successfully dissolved the microthrombi, whereas saline did not (Fig. 6). Injection of EACA 10 minutes before microthrombus formation prevented this tPA-evoked thrombolysis. To investigate how exogenously administered tPA can evoke fibrinolysis *in vivo*, Glu-plg-568 was given intravenously before laser injury and its accumulation was monitored in the GFP-mice treated with tPA after microthrombi formation. tPA administration evoked a sharp increase in Glu-plg-568 accumulation, which was followed by thrombolysis (Fig. 7A). The maximum Glu-plg-568 relative fluorescence intensities were higher after tPA infusion (3.6±1.14 fold, mean ± SD, n = 3) than after saline administration (1.15±0.33, mean ± SD, n = 3, P<0.05) (Fig. 7B). Taken together, these results clearly show that exogenously administered tPA successfully evoked plasminogen activation, resulting in a substantial increase in plasminogen accretion within the thrombus and also in the ultimate dissolution of the thrombus.

## Discussion

The results obtained in this study indicate that Glu-plg-568 accumulated in the center of a microthrombus in a time-dependent manner by binding to either the surface of activated platelets or to fibrin. The accumulation was LBS-dependent and required endogenously generated plasmin activity.

The activation of both platelets and the coagulation cascade is precisely regulated in a time- and space-dependent manner. We recently introduced a new approach in which the extent of platelet activation and the expression of procoagulant activity can be evaluated *in vivo* by detecting PS using fluorescent-labeled annexin A5 with an intra-vital imaging system [5]. We demonstrated that the extent of platelet activation is very different at various loci in the thrombus, and that only platelets existing in the center of the thrombus were fully activated to expose PS. The present study applies this technique to examine how and where the fibrinolytic system is triggered to dissolve microthrombi. We demonstrated that Glu-plg-568 accumulated in a time-dependent manner within 1 hour after laser injury in the center of the thrombus, where ANX-488 accumulated and GFP fluorescence intensity decreased after an initial increase. Thus, the site of plasminogen accumulation coincided with PS-exposing platelets and fibrin deposition. The time course of the accumulation was slower than those of PS exposure on the platelet surfaces (which reached a plateau at approximately 5 minutes) and fibrin formation (which reached a plateau at approximately 20 minutes) [5]. The accumulation appeared to be LBS-dependent, suggesting that the C-terminal lysine of either fibrin or other proteins localized on the platelet membrane could be the binding site.

Glu-plg is known to bind not only to fibrin but also to platelets through the fibrinogen receptor GPIIb/IIIa, especially when they are activated [13] [14]. The present *in vitro* study demonstrates that Glu-plg-568 binds to activated platelets in the absence of supplemented fibrinogen. The binding, however, was observed only after the disappearance of GFP, suggesting that the platelets were fully activated and that they exposed PS. Thus the presence of either extensively activated platelets or fibrin appeared to be a prerequisite for Glu-plg-568 accumulation on the platelet surface, which is in agreement with our *in vivo* results shown in the present study.

Aprotinin, a non-specific serine protease inhibitor that is known to efficiently inhibit plasmin activity, significantly suppressed Glu-plg accumulation in the microthrombus. As a chymotrypsin-type serine protease, plasmin cleaves peptide bonds with either a Lys or Arg residue at the P1 position, exposing C-terminal Lys residues in the substrate molecule after limited proteolysis. These newly exposed C-terminal lysines provide additional binding sites for Glu-plg and allow acceleration of fibrinolysis. Recently, by using an *in vitro* imaging system with CLSM, we demonstrated that Glu-plg accumulated in a time-dependent manner around GFP-tagged tPA (tPA-GFP) expressing EA.hy926 cells. This is a human vascular endothelial cell line, and the accumulation was significantly suppressed by aprotinin, *α*2-antiplasmin, or plasminogen activator inhibitor 1 (PAI-1) [4]. We also demonstrated that the fibrin network formed over the tPA-GFP expressing EA.hy926 cells was effectively lysed by secreted tPA-GFP. In the process of the lysis, the binding of Glu-plg-568 was continually observed on the lytic edge of the fibrin network, where the fibrin fiber is partially degraded. This binding was abolished by EACA, and it was negligible when catalytically non-active tPA-GFP-expressing EA.hy926 cells were employed [4]. Thus, the generation of C-terminal lysine residues by endogenously generated plasmin appears to be essential for both the binding of Glu-plg and for propagation of fibrinolysis [4]. Similarly, in an *in vivo* model of microthrombi formation, plasmin is likely to be endogenously generated. It will then trigger accumulation of Glu-plg-568 by enzymatic cleavage of proteins within the thrombus, exposing C-terminal lysines *de novo*. The tPA secreted either from intact VECs existing around the injury site, or attached to microparticles and circulating in plasma [15], may be involved in plasmin generation.

The thrombus generated in this model was stable and did not change appreciably in size during a 2-hour period of observation, despite the accumulation of Glu-plg in the thrombus over time. Intravenously administered therapeutic doses of tPA, but not of saline, however, increased the accumulation of Glu-plg and successfully dissolved the thrombus. Furthermore, EACA blocked this tPA-induced thrombolysis. These results suggest that tPA preferentially binds to the thrombus and effectively triggers plasminogen activation and thrombolysis [3] [16].

Because of its advantageous characteristics for effectively evoking fibrinolysis after specifically binding to fibrin, tPA is mainly used as a thrombolytic agent. Its usefulness is still limited, however, by insufficient delivery as well as side effects, including hemorrhage and central nervous system toxicity [17,18]. Our present finding that Glu-plg accumulates in the core of the microthrombus, where PS is exposed on platelet surfaces and fibrin formation takes place, should provide an extremely useful basis for the development of targeted and safer thrombolysis. This result argues for new approaches to improve the effectiveness of PAs by increasing affinity and specificity to thrombus, in which fibrin, platelets, platelet endothelial cell adhesion molecule-1 or glycophorin A on red blood cells have already been targeted to anchor PAs to the thrombus or the injured vascular lumen [19] [20] [21] [22]. Accordingly, the advantages of single chain urokinase—which is catalyzed to its active form by plasmin [23]—for use as an effective thrombus-targeted PA might be re-investigated, since plasmin activity was expressed at an early phase in the core of the thrombus.

Based on the finding that Glu-plg accumulated in the thrombus and that this accumulation depended on endogenously generated plasmin activity, it can be concluded that fibrinolysis starts in the microthrombus at an early phase, even when the size of the thrombus is unchanged. However, the plasmin activity generated in the early phase and the Glu-plg accumulation triggered in the thrombus were not strong enough to initiate thrombolysis. We believe that further elucidation of the mechanisms regulating this endogenous plasmin generation in the early phase of thrombus formation may suggest new approaches for the development of novel thrombolytic therapies.
